# DNA Polymerases as targets for gene therapy of hepatocellular carcinoma

**DOI:** 10.1186/s12885-015-1339-1

**Published:** 2015-04-29

**Authors:** Hao Liu, Qun Wei, Jia Wang, Xiaoming Huang, Chunchun Li, Qiaoli Zheng, Jiang Cao, Zhenyu Jia

**Affiliations:** 1 Institute of Occupational Diseases, Zhejiang Academy of Medical Sciences, 182 Tianmushan Road, Hangzhou, 310013 Zhejiang P.R. China; 2 Department of Surgical Oncology, Sir Run Run Shaw Hospital, Zhejiang University School of Medicine, 3 East Qingchun Road, Hangzhou, 310016 Zhejiang P.R. China; 3 School of Laboratory Medicine and Life Science, Wenzhou Medical University, Chashan Higher Educational Park, Wenzhou, 325035 Zhejiang P.R. China; 4 Clinical Research Center, The Second Affiliated Hospital, Zhejiang University School of Medicine, 88 Jiefang Road, Hangzhou, 310009 Zhejiang P.R. China

**Keywords:** Hepatocellular carcinoma, Gene therapy, Artificial microRNA, DNA polymerase, Caspase 3

## Abstract

**Background:**

Hepatocyte carcinoma (HCC) is one of the most common malignancies worldwide. Despite many achievements in diagnosis and treatment, HCC mortality remains high due to the malignant nature of the disease. Novel approaches, especially for targeted therapy, are being extensively explored. Gene therapy is ideal for such purpose for its specific expression of exogenous genes in HCC cells driven by tissue-specific promoter. However strategies based on correction of mutations or altered expressions of genes responsible for the development/progression of HCC have limitations because these aberrant molecules are not presented in all cancerous cells. In the current work, we adopted a novel strategy by targeting the DNA replication step which is essential for proliferation of every cancer cell.

**Methods:**

A recombinant adenovirus with alpha fetoprotein (AFP) promoter-controlled expressions of artificial microRNAs targeting DNA polymerases α, δ, ε and recombinant active Caspase 3, namely Ad/AFP-Casp-AFP-amiR, was constructed.

**Results:**

The artificial microRNAs could efficiently inhibit the expression of the target polymerases in AFP-positive HCC cells at both RNA and protein levels, and HCC cells treated with the recombinant virus Ad/AFP-Casp-AFP-amiR exhibited significant G0/1 phase arrest. The proliferation of HCC cells were significantly inhibited by Ad/AFP-Casp-AFP-amiR with increased apoptosis. On the contrary, the recombinant adenovirus Ad/AFP-Casp-AFP-amiR did not inhibit the expression of DNA polymerases α, δ or ε in AFP-negative human normal liver cell HL7702, and showed no effect on the cell cycle progression, proliferation or apoptosis.

**Conclusions:**

Inhibition of DNA polymerases α, δ and ε by AFP promoter-driven artificial microRNAs may lead to effective growth arrest of AFP-positive HCC cells, which may represent a novel strategy for gene therapy by targeting the genes that are essential for the growth/proliferation of cancer cells, avoiding the limitations set by any of the individually altered gene.

## Background

Hepatocellular carcinoma (HCC) is one of the most frequently diagnosed cancers and one of the leading causes of cancer death in both men and women worldwide, and HCC incidence rates are increasing in many parts of the world [1-4]. Despite of the achievements in early diagnosis and treatment, HCC mortality remains high due to its malignant nature. At present, surgical resection and liver transplantation remain to be the most curative treatment for early stage HCC. Nevertheless, patients recurrence is up to 70% within five years after surgical section; the strict surgical indications, limited liver donors and high costs restrict liver transplantation to only a minority of patients. Nonsurgical treatments include percutaneous ablation, transarterial chemoembolization, radioembolization and systemic chemotherapy [1,3-5].

As a new form for cancer treatment, gene therapy has been used for certain cancers, and a number of clinical trials including phase I, II and III trials for various cancers are underway [6,7]. Recent gene therapy for HCC is still confined to pre-clinical laboratory investigations, focusing on single or multiple genes dysregulated/mutated in HCC cells [8-12]. Gene therapy exhibits synergistic antitumor ability when combined with radiotherapy or chemotherapy [13-16], However, mutations or aberrant expressions of those target genes are highly variable in HCC, the strategy targeting one or a few of alterations may only be effective for a small group of patients. It is highly desirable to develop a novel strategy that will be effective for more, if not all, HCC.

DNA replication is one of the key steps for cell proliferation, and DNA polymerase is essential for DNA replication [17]. Inhibition of DNA polymerase expression by gene-silencing should therefore be sufficient to block the proliferation of cancer cells. Apoptosis, a natural biological progress which can be triggered by intrinsic mitochondrial pathway and extrinsic death receptor pathway [18,19], plays a crucial role in eliminating excess or abnormal cells and which is often impaired in cancer. Endogenous pro-Caspase 3 is unable to induce apoptosis, and Caspase 3 activity also determines the chemosensitivity of cancer cells [20]. Therefore we hypothesized that the combination of silencing DNA polymerases and enforcing expression of recombinant active Caspase 3 should have potent antitumor effect which may inhibit cell proliferation while trigger apoptosis.

In the current study, a combination of AFP enhancer and AFP basal promoter was adopted to modulate the HCC specific expression of artificial microRNAs targeting DNA polymerases α, δ and ε simultaneously with constitutively active recombinant Caspase 3 in an adenoviral vector. Serum alpha-fetoprotein (AFP) is extensively used as a tumor biomarker [21], and its promoter is therefore widely used to achieve HCC-specific gene expression with different enhancer/promoter combinations to confer high level while tight control of downstream gene expression in AFP-positive hepatoma cells [22-25]. The recombinant adenovirus presented in this work showed potent antitumor efficacy targeting AFP-positive HCC.

## Methods

### Cell culture

Human hepatocellular carcinoma cell lines HepG2 (ATCC, Manassas, VA, USA; B-8065) and Hep3B (ATCC, HB-8064) purchased from American Type Culture Collection (ATCC) were maintained in RPMI 1640 medium (Life Technologies, Carlsbad, CA, USA; 22400–105) supplemented with 10% heat-inactivated bovine growth serum (BGS) (Thermo Scientific, Waltham, MA, USA; SH30541.03). Human normal liver cell line HL-7702 (Shanghai Institute of Cellular Biology of Chinese Academy of Sciences, Shanghai, China; GNHu6) was maintained in RPMI 1640 medium with 10% heat-inactivated fetal bovine serum (FBS) (Life Technologies,10099-141). Human embryonic kidney cell line Adeno-X-293 (Clontech Laboratories, Mountain View, CA, USA; 632271) was maintained in low glucose Dulbecco’s Minimum Essential Medium (DMEM) (Life Technologies,12320-032) supplemented with 10% BGS. All cells were cultured at 37°C and 5% CO_2_ with saturated humidity and splitted when reach confluency.

### Generation of recombinant adenovirus

#### Construction of pDC312/AFP-Casp-AFP-amiR

**Figure 1:**
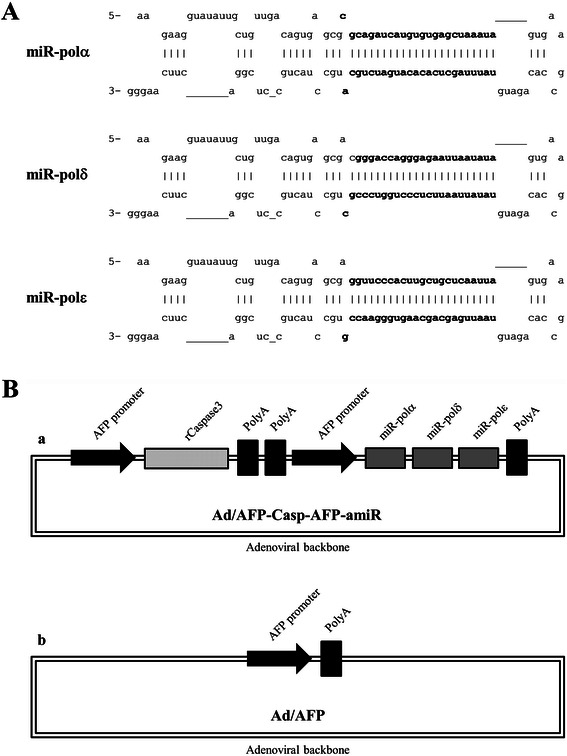
Illustration of hairpin structure of artificial microRNAs and recombinant adenoviruses. **A**: DNA sequences of artificial miRNAs targeting DNA polymerases α, δ and ε were based on natural structure of human miR-30. The bolds were core sequences acquired from RNAi Codex. **B**: a) Recombinant adenovirus Ad/AFP-Casp-AFP-amiR containing two expression cassettes to express artificial microRNAs targeting DNA polymerase α, δ, ε and active recombinant Caspase3. b) Recombinant adenovirus Ad/AFP only containing AFP promoter used as control.

A 1018 bp hepatocyte-specific recombinant AFP enhancer/promoter composed of the enhancer region from −4120 to −3300 and the basal promoter from −180 to +1 of human AFP gene was cloned as previously reported [26]. Constitutively active recombinant caspase 3 gene was cloned as previously described [27], and subcloned into adenoviral shuttle plasmid pDC312 (Microbix Biosystems, Toronto, ON, Canada) under recombinant AFP enhancer/promoter to form a Caspase 3 expression cassette as shown in Figure 1A. DNA sequences of artificial miRNAs targeting DNA polymerases α, δ and ε were based on natural structure of human miR-30 (miRBase accession number : MI0000088) and core sequences were acquired from RNAi Codex (http://cancan.cshl.edu/cgi-bin/Codex/Codex.cgi) (Figure 1A). These DNA fragments were cloned by two rounds of overhang extension PCR with cycling condition: 95°C, 2 min; 95°C, 30s, 58°C, 30s, 72°C, 30s, 30 cycles; 72°C, 5 min [28].Primers are listed in Table 1. Briefly, the 97 bp products of the first round PCR amplification were used as templates for the second round amplification, and the 142 bp final PCR products were cloned into pMD19-T (TaKaRa Bio, Otsu, Shiga, Japan; D102A) and sequenced by Invitrogen (Shanghai, China). Correct sequences were cloned into expression vector pIRES2-EGFP (Clontech Laboratories, 6029–1). The tandem repeats of artificial miRNAs were ligated into adenoviral shuttle vector pDC312 under recombinant AFP enhancer/promoter to form the artificial miRNAs expression cassette AFP-amiR, followed by the AFP-Casp expression cassette in the same orientation, as schematically depicted in Figure 1B. Transcription terminal signal was cloned from the BGH polyA of pcDNA3.1 (+) (Life Technologies, V790-20). The resulting shuttle vector was designated as pDC312/AFP-Casp-AFP-amiR. Another plasmid, pDC312/AFP, an empty vector without exogenous genes was constructed as control as schematically described in Figure 1B. Every two neighbouring fragments were ligated by *Bam*HI/*Bgl*II (New England Biolabs, Ipswich, MA, USA; R0136/R0144) cohesive ends [29].

**Table 1:** **PCR primers for amplification of artificial microRNAs**

artificial microRNA		primer sequence	product size(bp)
miR-polα	1st round	F: 5-TGCTGTTGACAGTGAGCGCGCAGATCATGTGTGAGCTAAATAGTGAAGCCACAGATGTA-3	97
		R: 5-TCCGAGGCAGTAGGCATGCAGATCATGTGTGAGCTAAATACATCTGTGGCTTCACTATT-3	
	2nd round	F: 5-***AGATCT***GATCCAAGAAGGTATATTGCTGTTGACAGTGAGCG-3	142
		R: 5-***GGATCC***ATCGTAGCCCTTGAAGTCCGAGGCAGTAGGCA-3	
miR-polδ	1st round	F: 5-TGCTGTTGACAGTGAGCGACGGGACCAGGGAGAATTAATATAGTGAAGCCACAGATGTA-3	97
		R: 5-TCCGAGGCAGTAGGCAGCGGGACCAGGGAGAATTAATATACATCTGTGGCTTCACTATA-3	
	2nd round	F: 5-***AGATCT***GATCCAAGAAGGTATATTGCTGTTGACAGTGAGCG-3	142
		R: 5-***GGATCC***ATCGTAGCCCTTGAAGTCCGAGGCAGTAGGCA-3	
miR-polε	1st round	F: 5-TGCTGTTGACAGTGAGCGAGGTTCCCACTTGCTGCTCAATTAGTGAAGCCACAGATGTA-3	97
		R: 5-TCCGAGGCAGTAGGCACGGTTCCCACTTGCTGCTCAATTACATCTGTGGCTTCACTAAT-3	
	2nd round	F: 5-***AGATCT***GATCCAAGAAGGTATATTGCTGTTGACAGTGAGCG-3	142
		R: 5-***GGATCC***ATCGTAGCCCTTGAAGTCCGAGGCAGTAGGCA-3	

The bold italics indicate restriction endonuclease sites for cloning.

### Packaging, characterization*,* propagation, purification and titration of recombinant adenovirus

The adenoviral shuttle plasmids pDC312/AFP-Casp-AFP-amiR and pDC312/AFP were cotransfected with adenoviral backbone plasmid pBGHlox(delta)E1,3Cre vector (Microbix Biosystems) by Lipofectamine LTX (Life Technologies, 11668–019) into human Adeno-X™ 293 cells respectively. Supernatant were analyzed to characterize the generation of recombinant adenoviruses performed by PCR amplification of inserted exogenous gene sequences. Primers and reaction conditions are listed in Table 2. The recombinant adenoviruses were propagated by infecting Adeno-X™ 293 cells with 5% serum DMEM in large scale and infected cells shown cytopathic effect (CPE) in 48 h were harvested. Virus particles were released by three rounds of freeze-thaw cycle at −80°C and 37°C and purified by Adeno-X Maxi Purification Kit (Clontech Laboratories, 631533) for in vitro assay. Purified viruses were dialyzed using by sterile Slide-A-Lyzer Dialysis (Thermo-Pierce, Rockford, IL, USA; 66453) with viral storage buffer (20 mM Tris.Cl pH8.0, 25 mM NaCl, 2.5% glycerol(w/v)). Infectious units (ifu) of dialyzed viruses were titrated by Adeno-X Rapid Titer Kit (Clontech Laboratories, 632250) following the manufacturer’s instruction. Titrated viruses were aliquoted and stored at −80°C.

**Table 2:** **PCR primers and reaction conditions for identification of recombinant adenoviruses**

gene detected	primer sequence	PCR reaction condition	product size(bp)
miR-polα-δ-ε	F: 5-***AGATCT***GATCCAAGAAGGTATATTGCTGTTGACAGTGAGCG-3	95°C , 5 min; 94°C , 30s, 50°C , 30s, 72°C , 45 s, 35 cycles; 72°C , 5 min	142, 284, 426
	R: 5-***GGATCC***ATCGTAGCCCTTGAAGTCCGAGGCAGTAGGCA-3		
polyA	F: 5-***AGATCT***TGTGCCTTCTAGTTGCCAGC-3	95°C , 5 min; 94°C , 30s, 53°C , 30s, 72°C , 30s, 35 cycles; 72°C , 5 min	237
	R: 5-***GGATCC***GCCATAGAGCCCACCGCATC-3		
AFP promoter	F: 5-***AGATCT***CAGATTGAATTATTTGC-3	95°C , 5 min; 94°C , 30s, 55°C , 30s, 72°C , 90s, 35 cycles; 72°C , 5 min	1018
	R: 5-***GGATCC***AAATCATGCTGAAATT-3		
recombinant caspase3	F: 5-***AGATCT***GGCTAACTAGAGAACCCA-3	95°C , 5 min; 94°C , 30s, 58°C , 30s, 72°C , 60s, 35 cycles; 72°C , 5 min	610
	R: 5-***GGATCC***CCCATCAACTTCATCGTGATAAAAATAGAGTTC-3		

The bold italics indicate restriction endonuclease sites for cloning.

### Cell viability assay by MTT

HepG2, Hep3B and HL7702 cells were seeded in 24-well cell culture plates with 1 × 10^4^ in 0.5 ml complete medium per well, and infected with multiplicity of infection (MOI) 50 by Ad/AFP-Casp-AFP-amiR and Ad/AFP respectively in triplicates. Cell viability was determined by MTT assay using thiazolyl blue tetrazolium bromide (Sigma, St. Louis, MO, USA; M2128). At designated time point post infection, the infected cells were incubated with MTT solution with a final concentration of 0.5 mg/ml for 4 h at 37°C, 5% CO_2_ and saturated humidity. Medium was aspirated, 250 μl isoproponal was added per well to dissolve purple crystals and 100 μl of the dissolved solution was transferred into a 96-well plate for measurement of absorbance at 570 nm with a 690 nm reference by Molecular Device Spectra Max M4 Microplate Reader. Relative cell viability was calculated with the non-infected cells as controls. Experiment was repeated at least twice.

### Flow cytometry analysis of cell apoptosis by Annexin V-PI staining

HepG2, Hep3B and HL7702 cells were seeded in 6-well cell culture plate with total number 6 × 10^5^, 8 × 10^5^ and 5 × 10^5^ respectively, and infected with recombinant adenoviruses at MOI 50 for 72 h. Infected cells were collected by centrifugation and washed with phosphate-buffered saline (PBS). Early apoptosis was detected by flow cytometry with FITC-Annexin V Apoptosis Detection Kit (BD Biosciences Pharmingen, San Diego, California, USA; 556547) following the manufacturer’s instruction. Experiment was repeated at least 3 times.

### Cell cycle examined by PI staining coupled with flow cytometry

HepG2, Hep3B and HL7702 cells were seeded in 6-well cell culture plate with total number 2 × 10^6^, 4 × 10^6^ and 1 × 10^6^ respectively, and infected with recombinant adenoviruses at MOI 50 for 48 h. Infected cells were collected by centrifugation, fixed in 75% pre-chilled ethanol at 4°C overnight and washed with PBS and stained by staining solution containing 50 μg/ml Propidium Iodide (PI) (Sigma, P4170) and 100 μg/ml RNase A (AndyBio, Itasca, IL, USA; A0051), 0.1% Triton X-100 (Amresco, Solon, OH, USA; 0694) at 37°C for 30 min in dark. Cell cycle profiles were analyzed by flow cytometry. Experiment was repeated at least three times.

### Real time quantitative PCR

HepG2, Hep3B and HL7702 cells were seeded in 6-well cell culture plate with total number 1.2 × 10^6^, 1.6 × 10^6^ and 5 × 10^5^ respectively, and infected with recombinant adenoviruses at MOI 50 for 48 h. Total RNA was extracted by RNeasy Plus Mini Kit (Qiagen, Hilden, Germany; 74134), and reverse transcribed to cDNA by M-MLV Reverse Transcriptase (Promega, Madison, WI, USA; M1701). Fluorescent real time PCR with QuantiFast SYBR Green PCR (Qiagen, 204054) was performed to examine the mRNA levels of AFP, DNA polymerase α, δ and ε, with β-Actin as a normalization control according to the manufacturer’s instruction. Triplicates were set up for each sample and experiment was repeated at least twice. Primers were listed in Table 3.

**Table 3:** **Quantitative PCR primers for determination of mRNA expression levels of polymerase**$\boldsymbol{\upalpha}$, $\boldsymbol{\updelta}$, $\boldsymbol{\upvarepsilon}$**, AFP and**$\boldsymbol{\upbeta}$**-actin**

gene detected	gene code	primer sequence	product size(bp)
polymerase α	NM_016937.3	F: 5- TTCTGACAAGTCCCTGTACACC −3	240
		R: 5- TAGGATTTCACGGCACAACCA −3	
polymerase δ	NM_002691.3	F: 5- CCCTACGTGATCATCAGTGC −3	107
		R: 5- AGGTAGTACTGCGTGTCAATGG −3	
polymerase ε	NM_006231.2	F: 5- CCTACGACTCCTCTGCCATC −3	219
		R: 5- CGTAGTGCTGGGCAATGTTC −3	
AFP	NM_001134.1	F: 5- ACCTCGTCGGAGCTGATGG −3	204
		R: 5- TGGCCTCCTGTTGGCATATG −3	
β-actin	NM_001101.3	R: 5- CCGATCCACACGGAGTACTT −3	406

### Western blotting

Cells were plated and infected with recombinant adenoviruses in the same manner as that for qPCR. Cells were harvested 48 h after infection and lysed. Protein concentration was determined by BCA protein assay kit (Thermo-Pierce, 23227) and samples were subject to SDS-PAGE followed by electrotransfer onto PVDF immobilon-P membrane (Merck Millipore, Billerica, MA,USA; IPVH00010). Antibodies against DNA polymerase α (SANTA CRUZ, Dallas, Texas, USA; sc-5921), DNA polylmerase δ (SANTA CRUZ, sc-8800), DNA polymerase ε (SANTA CRUZ, sc-56655), Caspase 3 (Cell Signaling Technology, Danvers, MA, USA; 9662S) and β-Actin (SANTA CRUZ; sc-47778) were used as primary antibodies. Specific bands were visualized by Clarify Western ECL Substrate (Bio-Rad Laboratories, Hercules, CA, USA; 170–5061) with Gel Imaging and System (Alpha Innotech Corporation, Randburg, South Africa; FC2) and relative protein expression level was calculated by AlphaVIEW SA software. β-Actin was probed on the same PVDF membrane as an internal control for protein equal loading. Experiment was repeated at least twice.

### Statistics

All data were presented as mean value ± standard deviation (SD). P-values between treatment and control groups were analyzed by unpaired Student’s t-test. *P* < 0.05 was considered statistically significant.

## Results

### Recombinant adenovirus inhibits the expression of DNA polymerases in HCC cells and decreases S-phase fraction

**Figure 2:**
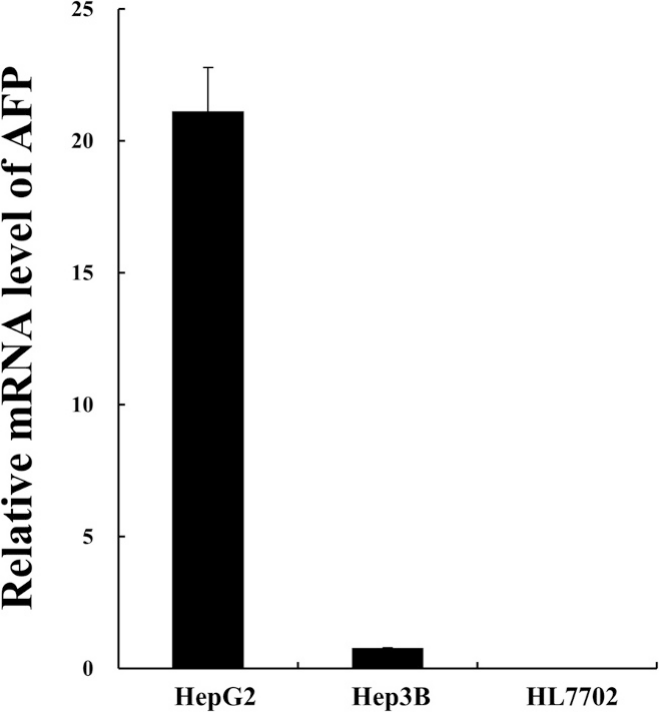
Relative expression level of AFP in three cell lines. AFP and β - actin mRNA levels of HCC cells HepG2, Hep3B and normal liver cell HL7702 were quantified by fluorescent real time quantitative PCR. β-Actin was used as the internal control to calculate the relative mRNA level of AFP.

**Figure 3:**
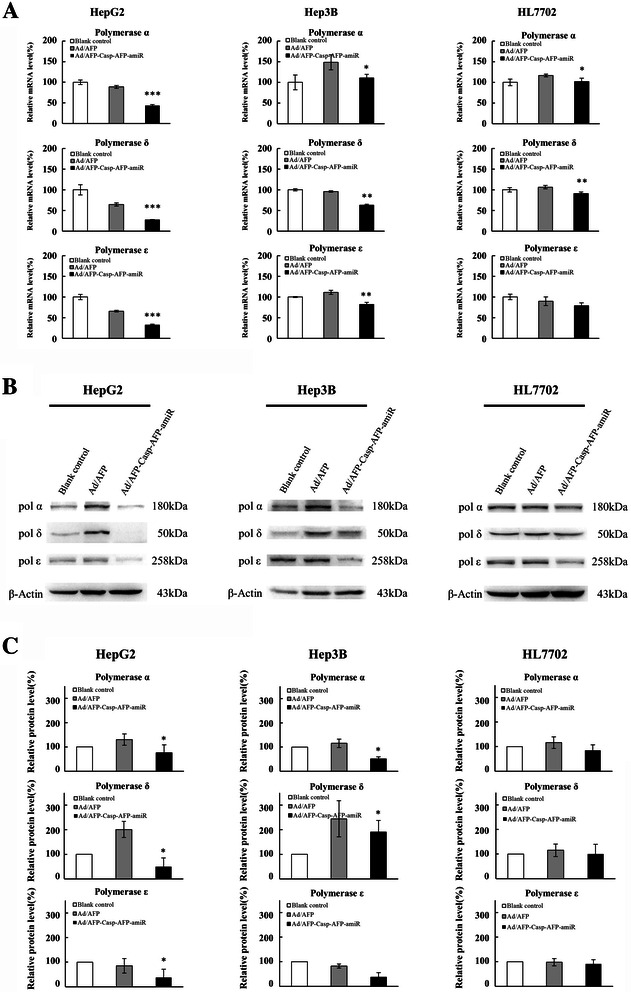
Ad/AFP-Casp-AFP-amiR inhibited expression of DNA polymerases in HCC cell lines. **A**: Ad/AFP-Casp-AFP-amiR inhibited mRNA expression of DNA polymerases in HCC cell lines. DNA polymerase α, δ, ε and β-actin mRNA levels of HCC cells HepG2, Hep3B and normal liver cell HL7702 were quantified by fluorescent real time quantitative PCR after infected by two adenoviruses with MOI 50 respectively for 48 h. β-Actin was used as the internal control to calculate the relative mRNA levels of DNA polymerase α, δ, ε. The relative mRNA level of blank control was set as 100%. *P < 0.05, **P < 0.01, ***P < 0.001, n = 3. **B**: Ad/AFP-Casp-AFP-amiR inhibited protein expression of DNA polymerases in HCC cell lines. DNA polymerase α, δ, ε and β-actin protein levels of HCC cells HepG2, Hep3B and normal liver cell HL7702 were monitored by Western blot after infected by two adenoviruses with MOI 50 respectively for 48 h. **C**: The grey values of the DNA polymerase α, δ, ε and β-actin were calculated by AlphaVIEW SA software. β-Actin was used as the internal control to calculate the relative protein levels of DNA polymerase α, δ, ε. The relative mRNA level of blank control was set as 100%. *P < 0.05, **P < 0.01, ***P < 0.001, n = 3.

The recombinant adenovirus Ad/AFP-Casp-AFP-amiR was constructed with two separate expression cassettes which express active Caspase 3 and tandem artificial microRNAs targeting DNA polymerases α, δ and ε respectively as illustrated in Figure 1B. Recombinant adenovirus Ad/AFP was constructed as control. Two HCC cells HepG2 and Hep3B and one normal hepatocyte cell HL7702 with different AFP expression levels were used to evaluate the targeted expression. As shown in Figure 2, high level of AFP mRNA could be detected by reverse transcription quantitative PCR (RT-qPCR) in HepG2 cells and low level of AFP mRNA could be detected in Hep3B cells. Relatively low level of AFP mRNA compared to those in HepG2 and Hep3B cells was detected in HL7702 cells (considered as AFP-negative). AFP level in HepG2 was ~ 26 folds higher than that in Hep3B, ~ 5 × 10^5^ folds higher than that in HL7702. We first examined the inhibition efficiency of artificial microRNAs on target mRNAs in these cells 48 h after infection by the recombinant adenovirus Ad/AFP-Casp-AFP-amiR. The results showed that only very weak inhibition in AFP-negative HL7702 cells could be observed, with inhibition rates 12.71% (p < 0.05, n = 3), 14.87% (p < 0.01, n = 3) and 12.06% (p > 0.05, n = 3) for DNA polymerases α, δ and ε respectively. Higher inhibition could be observed in Hep3B cells which have low AFP expression, with inhibition rates 25.51% (p < 0.05, n = 3), 34.33% (p < 0.01, n = 3) and 26.28% (p < 0.01, n = 3) for DNA polymerases α, δ and ε respectively. Strong inhibition was observed in HepG2 cells which have high level of AFP expression, with inhibition rates 51.19% (p < 0.001, n = 3), 58.27% (p < 0.001, n = 3) and 51.50% (p < 0.001, n = 3) for DNA polymerases α, δ and ε respectively (Figure 3A). Western blot results (Figure 3B, C) also showed that significant decrease of DNA polymerases α, δ and ε at protein level in AFP-expressing HCC cells, with decrease rates 42.91% (p < 0.05, n = 3), 75.91% (p < 0.05, n = 3) and 61.03% (p < 0.05, n = 3) respectively in HepG2, 54.32% (p < 0.05, n = 3), 20.58% (p < 0.05, n = 3) and 53.96% (p > 0.05, n = 2) respectively in Hep3B, but the rates were only 22.88% (p > 0.05, n = 3), 14.53% (p > 0.05, n = 3) and 6.68% (p > 0.05, n = 3) respectively in HL7702, which were consistent with the inhibitions at mRNA level.

**Figure 4:**
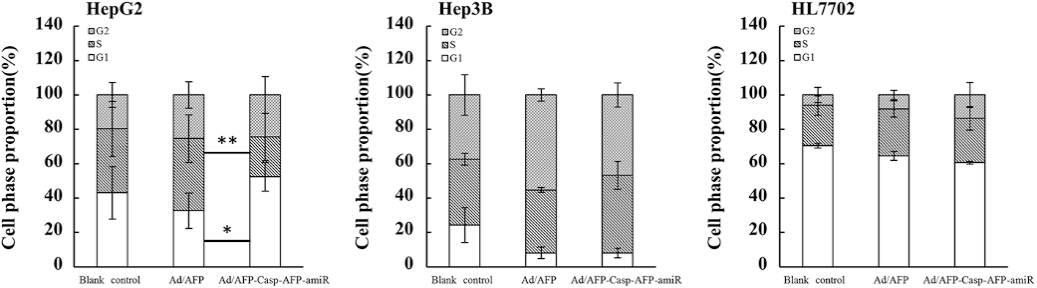
Ad/AFP-Casp-AFP-amiR decreased S phase in HepG2. Cell phase proportions of HCC cells HepG2, Hep3B and normal liver cell HL7702 were tested by PI staining with flow cytometry after infected by two adenoviruses with MOI 50 respectively for 48 h. *P < 0.05, **P < 0.01, n ≥ 3.

As a consequence of the expression inhibition of DNA polymerases by specific artificial microRNAs in HCC cells, the progression of cell cycle were significantly blocked. Flow cytometric cell cycle analyses showed retardant cell cycle. After infecting for 48 h, G1 phase increased significantly by 61.14% (p < 0.01, n = 6) and S phase decreased significantly by 44.91% (p < 0.05, n = 6) in high AFP-expressing HepG2 cells, while no statistically significant alterations were observed for low AFP-expressing Hep3B cells and AFP-negative HL7702 cells (Figure 4 and Table 4).

**Table 4:** **Influence of recombinant adenoviruses on S-phase Fraction (SPF) of HCC cells**

	S-Phase fraction			P-value
	Blank control	Ad/AFP	Ad/AFP-amiR-AFP-Casp	
HepG2	37.25%	42.00%	23.14%	<0.01
Hep3B	38.51%	36.55%	45.19%	>0.05
HL7702	23.40%	27.21%	25.69%	>0.05

P-values between Ad/AFP-Casp-AFP-amiR group and Ad/AFP group were analyzed by unpaired Student’s t-test.

### Recombinant adenovirus increases active Caspase 3 in HCC cells and promotes early apoptosis

**Figure 5:**
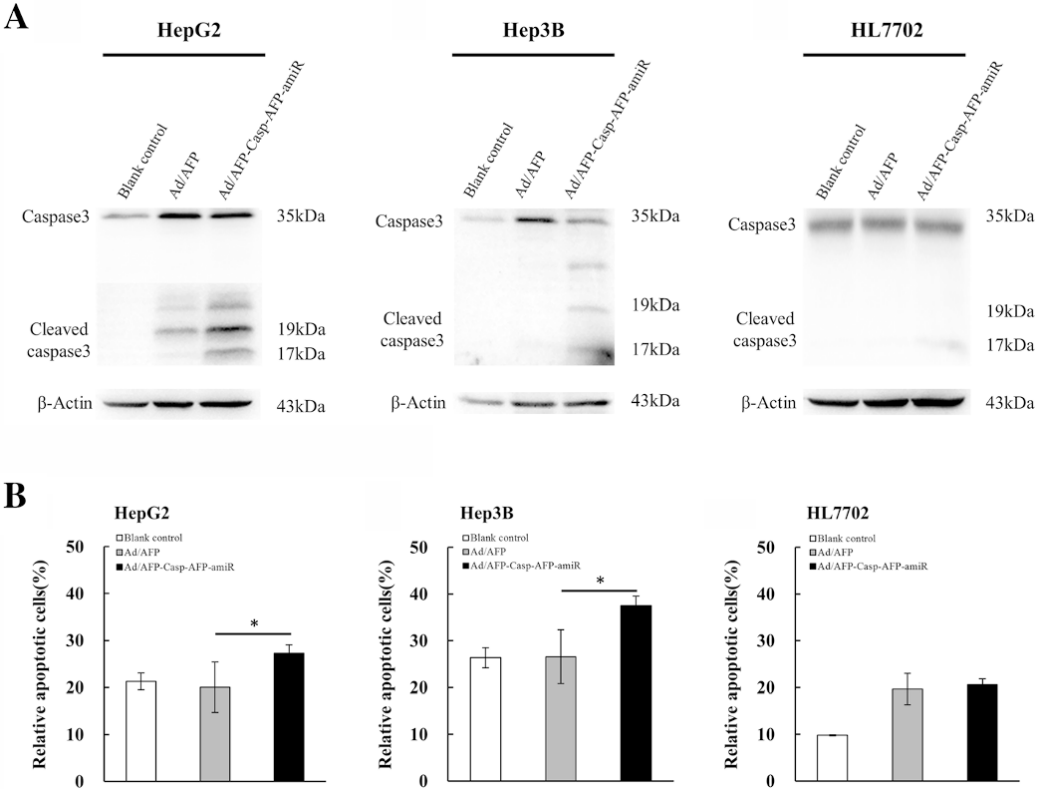
Ad/AFP-Casp-AFP-amiR induced cell apoptosis in HCC cell lines. **A**: Ad/AFP-Casp-AFP-amiR increased protein expression of cleaved Caspase3 in HCC cell lines. Caspase3 and β-actin protein levels of HCC cells HepG2, Hep3B and normal liver cell HL7702 were monitored by Western blot after infected by two adenoviruses with MOI 50 respectively for 48 h. **B**: Ad/AFP-Casp-AFP-amiR induced cell apoptosis in HCC cell lines. Relative apoptotic cells of HCC cells HepG2, Hep3B and normal liver cell HL7702 were determined by Annexin V staining coupled with flow cytometry after infected by two adenoviruses with MOI 50 respectively for 72 h. *P < 0.05, n = 4.

The HepG2, Hep3B and HL7702 cells were treated with the recombinant adenovirus Ad/AFP-Casp-AFP-amiR for 48 h and analyzed for caspase activation by Western blot. As shown in Figure 5A, Ad/AFP-Casp-AFP-amiR treatment significantly increased the activated effector Caspase 3 in HepG2 and Hep3B cells, suggesting effective activation of endogenous caspase cascade by the expression of recombinant active Caspase 3.

The specific activation of endogenous Caspase 3 in AFP-positive HCC cells by the recombinant adenovirus Ad/AFP-Casp-AFP-amiR led to the apoptosis of HCC cells. After infecting HCC cells for 72 h, Ad/AFP-Casp-AFP-amiR promoted early apoptosis by 35.97% (p < 0.05, n = 4) in HepG2, 41.15% (p < 0.05, n = 4) in Hep3B and 4.66% (p > 0.05, n = 4) in HL7702 respectively by Annexin V staining followed by flow cytometric examination, as shown in Figure 5B.

**Figure 6:**
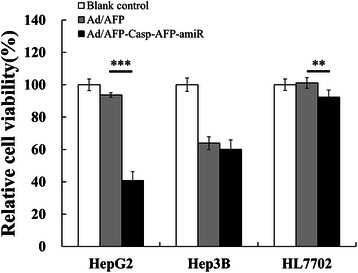
Ad/AFP-Casp-AFP-amiR inhibited proliferation of HepG2. Relative cell viabilities of HCC cells HepG2, Hep3B and normal liver cell HL7702 were detected by MTT assay after infected by two adenoviruses with MOI 50 respectively for 72 h. The relative cell viability was the ratio of treatment to control. **P < 0.01, ***P < 0.001, n = 5.

Cell viability assay by MTT also showed significant specific antitumor potential of Ad/AFP-Casp-AFP-amiR adenovirus in vitro. After infection for 72 h with MOI 50, compared to control virus Ad/AFP, Ad/AFP-Casp-AFP-amiR inhibited HepG2 cell survival by 56.40% (p < 0.001, n = 5), Hep3B by 5.90% with no significant difference and HL7702 by 8.72% (p < 0.01,n = 5) respectively (Figure 6).

## Discussion

One of the major problems in current cancer gene therapy is the side-effect caused by non-specific expression of exogenous genes in other cells than cancer cells. Tumor-specific promoter is a strategy commonly adopted in many studies nowadays. Since AFP gene is re-expressed in most HCC cells, its promoter is used as a regulatory element for HCC-specific expression. Though highly specific, the basal AFP promoter is weak in transcription initiation to achieve satisfactory therapeutic result. Therefore, an enhancer is often used in combination with AFP basal promoter for higher transcription initiation. Up to now, several enhancer-promoter combinations have been documented to attain potent and specific transcription in HCC gene therapy studies, such as the hypoxia-specific enhancer in combination with the AFP basal promoter [22], the AFP enhancer in combination with other promoter such as the housekeeping gene phosphoglycerate kinase (pgk) [23], and the AFP enhancer in combination with AFP basal promoter [24,25]. All these combinations exhibited HCC specific activity. High level expression can be achieved by the combination of AFP enhancer with its basal promoter which has shown high efficiency comparable to that of the most widely used non-specific cytomegalovirus (CMV) promoter [30]. Its high HCC-specific transcriptional activity is ensured by hepatocyte nuclear factors 1(HNF1), C/EBP, HNF3 and HNF4 binding to several cis-acting liver-enriched transcription factors (LETFs) binding sites in AFP enhancer [21,30]. In our study, a recombinant 1018 bp AFP promoter comprised of the enhancer region from −4120 to −3300 and the basal promoter from −180 to +1 of human AFP gene showed high HCC-specific activity [26], that the specific transcriptional factors/activators could bind to those specific binding sites in AFP enhancer and basal promoter to activate transcription of the downstream genes.

Effective therapeutic gene selection is another important issue needs to be considered in cancer gene therapy. Various aberrant genes in malignant cells have been targeted by different strategies to suppress the over-expressed genes or to compensate/enforce the expression of deleted/down-regulated genes. However, one gene may not work alone, it may be involved in more than one signaling pathways with interactions. One phenotype of a cell may be regulated by many genes, and the expression of one gene may determine many different aspects of a cell. When one gene is targeted, several interacting signal pathways may respond with feedback modulations. Therefore, targeting one to two individual aberrant genes in cancer cells may not lead to the expected results. As cells proliferate through cellular duplication including DNA replication [31], the indispensable DNA polymerases for DNA replication are ideal targets for gene therapy. DNA polymerases α, δ and ε that are responsible for DNA replication were chosen as our targets, which belong to polymerase family B and that Pol α initiates onset of DNA replication, followed by Pol ε and Pol δ which catalyzes leading and lagging strand synthesis respectively [32]. Artificial microRNA strategy was adopted in the study to mimic the knockdown of target gene as natural miRNAs do [33,34]. Previous reports showed that the processing of artificial pre-miRNAs to artificial mature miRNAs was more efficient when artificial pre-miRNAs were in tandem repeats than individual pre-miRNA, and the processed artificial mature miRNAs further led to more efficient inhibition on genes expression [29,35]. Therefore, to achieve better gene silencing, DNA sequences coding for artificial pre-miRNAs specifically targeting DNA Pol α, δ and ε were cloned in the same expression cassette (the artificial pre-miRNAs were simplified as artificial miRNAs in this paper). Transient transfection assay in Hep3B cells confirmed that the linearly-arrayed artificial miRNAs miR-polα-δ-ε expression vector was more potent in inhibiting Pol α, δ and ε at both mRNA and protein levels than each of the individual artificial miRNA expression vector (data not shown). Cancer cells may remain at quiescent state but not go apoptosis if only DNA replication is blocked by silencing of DNA polymerases. Caspase 3 is the key apoptosis executor responsible for a serial of substrates proteolytic degradation for mitochondrial apoptosis [27]. The strategy that expressing recombinant active Caspase 3 in combination with silencing DNA pol α, δ and ε can elicit significant therapeutic effect. In this work, the recombinant active Caspase 3 and artificial miRNAs were put in two expression cassettes separately with recombinant AFP enhancer/promoter and transcription termination signal bovine growth hormone polyadenylation (BGH poly A). An extra BGH poly A signal was placed between the two expressing cassettes to warrant the complete termination of AFP-Caspase 3.

Our current study showed the expected AFP-dependent inhibition of the recombinant adenovirus Ad/AFP-Casp-AFP-amiR on HCC cells. As the expression level of AFP is not constant in different HCC cells depending on the transcription efficiency of AFP promoter in a cell-specific manner, the proliferation-inhibition and apoptosis-inducing effects of the recombinant virus Ad/AFP-Casp-AFP-amiR differed in HepG2 and Hep3B cells due to different transcription efficiency of the recombinant Caspase 3 and artificial microRNAs controlled by AFP promoter in these two cells. For the minor inhibition observed for the control group, it might be an addition effect of viral toxicity and leakage expression of exogenous genes. Further assessments on the therapeutic value of our current strategy by *in vivo* experiments with HCC xenograph mouse models are needed in future work.

## Conclusions

In summary, the results from current work exhibited the highly efficient HCC-specific killing potential of the recombinant adenovirus Ad/AFP-Casp-AFP-amiR by the combination of HCC-specific AFP enhancer/promoter, blocking of DNA replication and triggering apoptosis. This may provide a novel strategy to HCC gene therapy.
